# Tuning Biodegradation of Poly (lactic acid) (PLA) at Mild Temperature by Blending with Poly (butylene succinate-co-adipate) (PBSA) or Polycaprolactone (PCL)

**DOI:** 10.3390/ma17225436

**Published:** 2024-11-07

**Authors:** Dimitri Van de Perre, Lynn Serbruyns, Maria-Beatrice Coltelli, Vito Gigante, Laura Aliotta, Andrea Lazzeri, Ruben Geerinck, Steven Verstichel

**Affiliations:** 1 Normec OWS nv, Pantserschipstraat 163, 9000 Ghent, Belgium; dimitri.van.de.perre@normecgroup.com (D.V.d.P.); lynn.serbruyns@normecgroup.com (L.S.); 2 Department of Civil and Industrial Engineering, University of Pisa, 56122 Pisa, Italy; vito.gigante@unipi.it (V.G.); laura.aliotta@unipi.it (L.A.); andrea.lazzeri@unipi.it (A.L.); 3 National Interuniversity Consortium of Materials Science and Technology (INSTM), 50121 Florence, Italy; 4 Centexbel, Technologiepark 70, 9052 Ghent, Belgium; rg@centexbel.be

**Keywords:** PLA, PBSA, PCL, biodegradation, plastic blend, mild temperature, home composting, marine, freshwater

## Abstract

Biobased plastics are fully or partially made from biological resources but are not necessarily biodegradable or compostable. Poly (lactic acid) (PLA), one of the most diffused bioplastics, is compostable in industrial environments, but improving degradation in home composting conditions, in soil and in seawater could be beneficial for improving its end of life and general degradability. Blends obtained by the extrusion of PLA with different amounts of poly (butylene succinate-co-adipate) (PBSA) or poly (caprolactone) (PCL) were characterized in terms of their home composting, soil, marine and freshwater biodegradation. The blending strategy was found to be successful in improving the home compostability and soil compostability of PLA. Thanks to the correlations with morphological characterization as determined by electron microscopy, it was possible to show that attaining an almost co-continuous phase distribution, depending on the composition and melt viscosity of the blend components, can enhance PLA degradation in home composting conditions. Tests in marine and freshwater were also performed, and the obtained results showed that in marine conditions, pure PLA is degradable. A comparison of different tests evidenced that salt dissolved in marine water plays an important role in favoring PLA’s degradability.

## 1. Introduction

Thanks to their low cost, good mechanical properties and versatility, synthetic polymers (e.g., plastics) have become an indispensable part of our lives and are used in numerous industrial and consumer products [1]. With an approximate production of 400 million metric tons per year, plastics are traditionally produced using materials (e.g., carbon, hydrogen, chloride, silicon and oxygen) extracted from oil, coal and natural gas [1,2]. However, the single-use culture and the intensive use of petrochemical-based polymers during the past few decades have resulted in huge non-degradable waste accumulation (e.g., in landfills and aquatic ecosystems), which has resulted in many negative environmental consequences. Williams et al. (2022) came to the conclusion that of all plastics discarded since 1952, 91% have never been recycled, ending up in environmental ecosystems or being dumped in landfills [3]. Currently, only 14% of all plastic is recycled, whereas only 2% is optimally recycled; the remaining 12% is downcycled. The OECD estimated that 22 million tonnes of plastic leaked into the environment in 2019 [4]. This value is projected to double, reaching 44 Mt by 2060. This plastic debris could have a negative impact (e.g., by ingestion, entanglement, light blocking or releasing toxic additives) on terrestrial and aquatic communities and ecosystems [5,6].

With the ever-increasing use of plastics worldwide, the strain on the environment and non-renewable natural resources will also keep increasing, eventually reaching critical levels. While the material recycling of plastics could, in theory, be the best solution to these problems, the high price and lower quality of recycled plastics (especially the ones coming from commingled or multimaterial plastics) limit their market applications [7,8]. The use of biodegradable plastics could limit the environmental impacts of plastic wastes, and when these biodegradable plastics are based on renewable resources, they could also address the threat of oil depletion [1,8,9]. These biodegradable plastics are especially useful for food packaging applications, where they can offer an additional recycling route via industrial composting and simultaneously result in a higher biowaste capture of spoiled food and lower the contamination of compost caused by the current non-biodegradable packaging applied. Furthermore, they can be valuable alternatives to current non-degradable materials for applications in the open environment that should degrade, e.g., agricultural mulch films and cropping twines, lawn trimmer threads, tree shelters, seed coatings, leg bands, plastic parts in fireworks, geotextiles, etc. [10]. Biodegradable plastics that degrade across a wide range of managed and unmanaged environmental conditions offer greater economic and environmental rewards compared to those that are limited to biodegradation in a narrower set of environments [8].

Polylactic acid (PLA) is one of the major biobased plastics (mainly produced from starch harvested from corn) and is used as a food packaging material, in tableware, consumer goods, textiles and in the agricultural industry [11]. PLA is in parity with other conventional plastics such as PP and PET in terms of various properties rendering it suitable for industrial usage, such as in terms of its mechanical and physical properties, biocompatibility and processability [8,12,13]. Additionally, a major advantage of PLA is the fact that it is biodegradable; however, this is not applicable in every environment [11]. The process of biodegradation refers to degradation based on microorganisms (bacteria, fungi and algae), where a polymer material (e.g., a plastic) is used as an energy source and is converted into CO_2_, H_2_O, minerals and biomass by biological processes [14]. PLA biodegradation is normally triggered by temperatures above 50 °C, and as such, it will degrade under industrial composting conditions but not at mild temperatures such as those in soil and sewage [13]. Furthermore, PLA has some drawbacks such as low toughness, low flexibility, brittleness and low thermal stability [8,15,16]. For example, above 58 °C, PLA becomes rubbery, and since PLA has a low glass transition temperature, it is not suitable for storing hot liquids [11]. One of the most effective methods of improving PLA’s mechanical properties (e.g., reduce brittleness) is to physically blend it with suitable biodegradable polyesters [8,16].

In general, the strategy of blending different polymers together is implemented in industry to combine, in a synergic way, their characteristics and therefore alter the functionality of their applications. In recent research, several different biobased and/or biodegradable polymers were successfully blended with PLA, altering its properties [8,15,17]. Poly (butylene succinate-co-adipate) (PBSA), a biobased polymer that is synthesized either by polycondensation from 1,4-butanediol with succinic acid and adipic acid or by transesterification from 1,4-butanediol and dimethyl esters of succinic acid and adipic acid, can be a suitable polymer for blending with PLA [18,19]. It is biodegradable and has good properties, such as high flexibility, thermal and chemical resistance and good impact strength [20].

Several studies have already illustrated that PLA shows good compatibility with PBSA. The blending of PBSA with PLA improves its toughness (due to the rubber toughening mechanism) and ductility [15,21]. These blends have been found to be suitable for producing recyclable biobased films [22]. Additionally, these studies have illustrated that it is possible to tailor the properties of PLA blends by controlling and adapting the blend compositions. PBSA was reported to be degradable in compost [23], in soil [24] and in seawater [25]. Another successful blending partner of PLA is Polycaprolactone (PCL) [8,16,26,27]. PCL is a petrochemical-based and biodegradable polymer that has good elongation, thermal and mechanical properties. When compared to the properties of pure PLA, PLA/PCL blends show, for example, an increase in the elongation at break, melting temperature toughness, and loss factor and a decrease in stiffness [8,16,26,27]. For example, the 80/20 PLA/PCL blend exhibited the highest elongation at break and its stiffness was 2.7-fold lower than for PLA alone [26]. PCL is reported to be degradable in compost at 28 °C [8] and at least partially degradable in natural water and seawater [28]. In the context of PLA blend biodegradation, Narancic et al. (2018) found that the 80/20 PLA/PCL blend degraded under home composting conditions (ISO 14855 at 28 °C) [8]. This result was very surprising since PLA alone is not home-compostable as its biodegradation normally requires a temperature trigger above 50 °C [13]. Thus, it could be deducted that producing blends can be an effective strategy for altering polymer biodegradability, and PLA can be made biodegradable at mild-temperature conditions. Therefore, to test whether this phenomenon also occurs at different compositions, we investigated the fate of a range of different PLA/PCL blend compositions under controlled home-compostable conditions. Although Narancic et al. (2018) demonstrated that blending PLA with PBS and PHB did not result in the same synergistic effect with regard to biodegradation at mesophilic temperatures, the present study investigated if blending PLA with PBSA could also result in an improved biodegradation behavior at mild temperatures [8]. The results are discussed on the basis of the polymer features and phase morphology of materials to attain useful correlations for their tailored formulation and processing.

## 2. Materials and Methods

### 2.1. Materials

Regarding PLA, commercial Ingeo 4043D (NatureWorks, Plymouth, MN, USA) was used for blending with PCL and Luminy LX175 (TotalCorbion, Gorinchem, The Netherlands) for blending with PBSA. Neat polymers (PLA, PCL and PBSA) (Table 1) were used to produce blends (Table 2) by twin-screw extrusion. PLA/PCL blends were prepared using a twin-screw extruder (Leistriz micro 27 compounder L = 120 mm/D = 27 mm). PLA LX175 from Total Corbion was blended with PBSA FD92PM purchased from Mitsubishi Chemical Corporation (Chiyoda City, Japan). Pure PLA LX175 and different ratios of PLA/PBSA were extruded in a semi-industrial Comac EBC 25HT (L/D = 44) (Comac, Cerro Maggiore, Italy) twin-screw extruder. Before the extrusion, all solid materials were dried in a Piovan DP 604-615 dryer (Piovan S.p.A., Verona, Italy). The temperature profile of the extruder (11 zones) used for blend preparation was 150/175/180/180/180/185/185/185/185/185/190 °C, with the die zone at 190 °C for all the blends except the one containing 40 wt.% of PBSA and pure PLA LX175. Due to the high quantity of PBSA that decreased the viscosity, for the blends containing 40 wt.% of PBSA, a slight decrease (5 °C) in temperature was applied: 150/170/175/175/175/180/180/180/180/180/185 °C, with the die zone at 185 °C. The extruded strands were cooled in a water bath at room temperature and reduced in pellets with an automatic knife cutter. All pellets were finally dried again at 60 °C. MFR and MVR values were measured with a CEAST (Torino, Italy) Melt Flow Tester INSTRON M20 equipped with an encoder. The ISO1133D standard was adopted: the sample was preheated without weight for 40 s at 190 °C, and then a weight of 2.160 kg was released on the piston, and after 5 s, a blade cut the spindle starting the real test (1 min). Through the encoder, every 3 s, one MVR measurement was recorded. The material flowing through the orifice was weighed, and the MFR value was obtained. Each polymer was preliminarily dried in an oven at 60 °C for one day before the tests. Before the additions to biodegradation reactors, the pellets were cryogenically milled (<1 mm).

### 2.2. Biodegradation Test Methods

**Figure 1:**
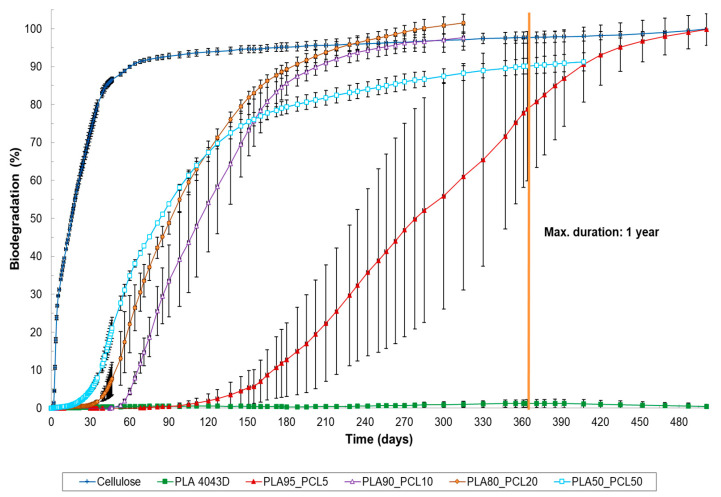
Biodegradation of cellulose (=reference material), PLA 4043D and several PLA/PCL plastic blends under home-composting conditions (ISO 14855, 28 °C). PCL is not reported here, but complete biodegradation of PCL was established in another home-composting biodegradation test.

**Figure 2:**
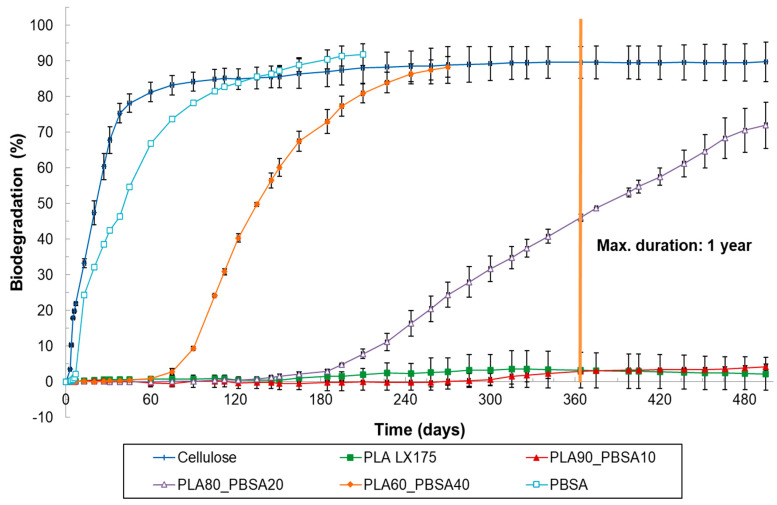
Biodegradation of cellulose (=reference material), PLA LX175, PBSA and several PLA/PBSA plastic blends under home-composting conditions (ISO 14855, 28 °C).

**Figure 3:**
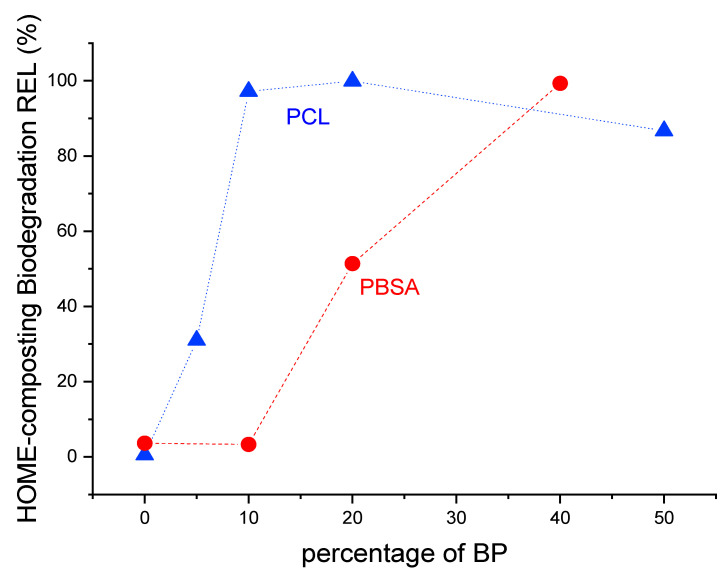
Dependency of the relative biodegradation of PLA blends from the content of a biodegradable polymer (BP) in home-composting conditions.

**Figure 4:**
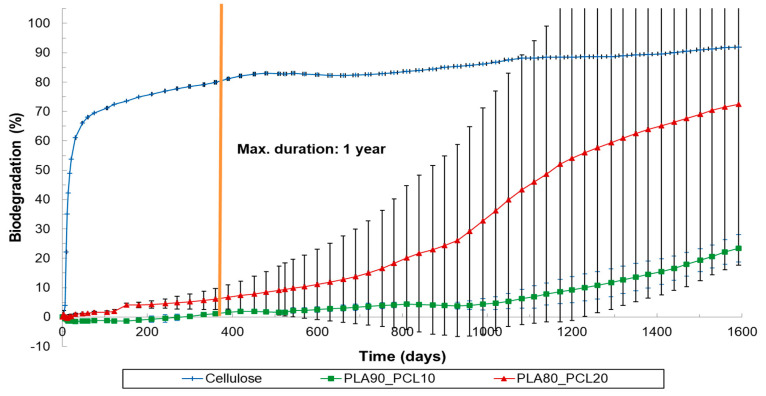
Biodegradation of cellulose (=reference material), PLA90_PCL10 and PLA80_PCL20 under soil conditions (ISO 17556, 25 °C).

**Figure 5:**
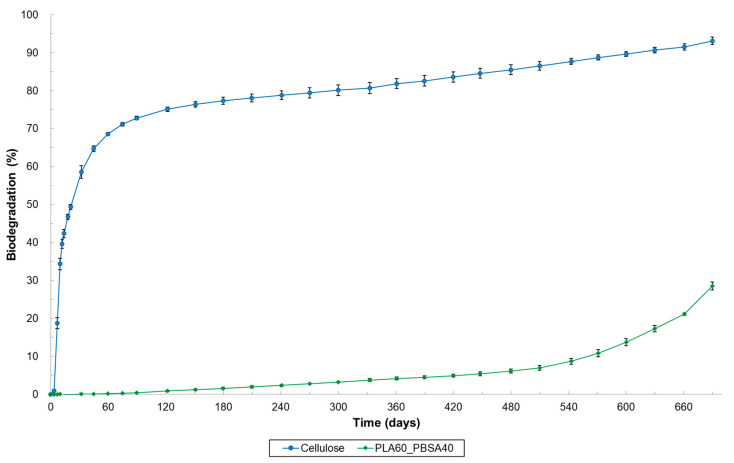
Biodegradation of cellulose (=reference material) and PLA60_PBSA40 plastic blend under soil conditions (ISO 17556, 25 °C).

**Figure 6:**
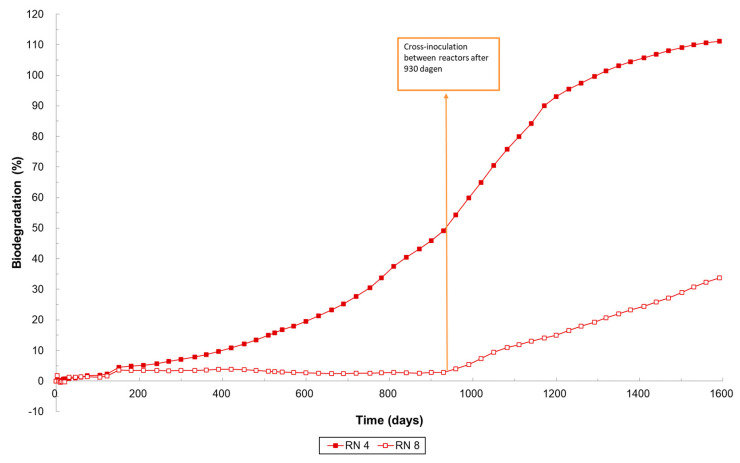
Biodegradation of replicates of PLA90_PCL20 under soil conditions (ISO 17556, 25 °C).

**Figure 7:**
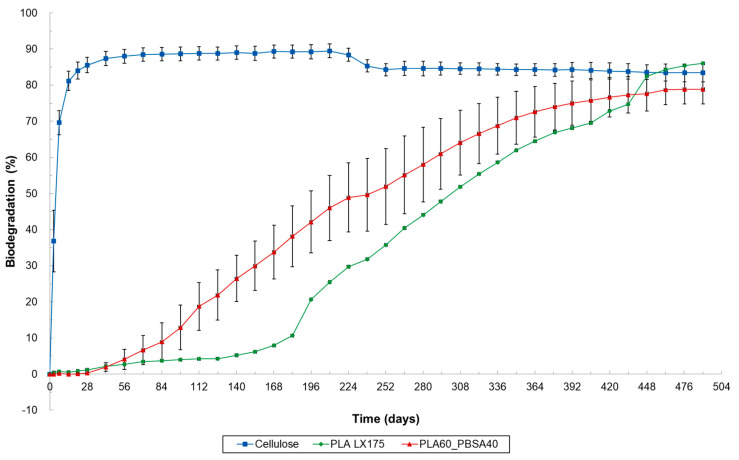
Biodegradation of cellulose (=reference material), PLA LX175 and PLA60_PBSA40 plastic blend under marine pelagic conditions (ASTM D6691, 30 °C; only one replicate for PLA LX175).

All the biodegradation tests were conducted according to international standards. A short description for each test is given below. For more details, see the different international standards used. Each test consisted of at least 2 replicates, a control (=no test item added) and a reference item (microcrystalline cellulose) to check the validity of the test. In the graphs of Figures 1, 2, 4 and 5, the standard deviation is shown, except for Figure 3 (reporting the final average value); Figure 6, which shows the different replicates; and Figure 7, for which one replicate of PLA LX175 had to be removed due to a technical problem. For some tests, the replicates showed a high deviation during the test. This is not unusual for these long-running natural processes. In fact, microbial adaptation can differ from reactor to reactor, especially for materials that are difficult to degrade under the tested conditions. The standard deviation decreases when the final biodegradation percentage is reached as the rate might differ during the test, but the final biodegradation percentage is generally similar. Moreover, sometimes, slight negative biodegradation percentages were obtained. This can be explained by a temporary higher activity in the control reactors compared to the test reactors, resulting in a negative net CO_2_ and, consequently, a biodegradation below zero. Similarly, a biodegradation percentage above 100% can occur and is caused by a synergistic effect, also called priming. A priming effect occurs if the inoculum in the test reactor is producing more CO_2_ than the compost inoculum in the control reactors. This results in a net CO_2_ production that is not exclusively coming from the test item, resulting in a biodegradation percentage of more than 100%. The duration of the experiments was sometimes adapted to investigate further developments over time.

#### 2.2.1. Home-Composting Biodegradation (ISO 14855 at 28 °C)

International standard ISO 14855 (Determination of the ultimate aerobic biodegradability of plastic materials under controlled composting conditions—Method by analysis of evolved carbon dioxide) (2012) was followed to conduct composting biodegradability tests on samples under dry, optimized aerobic conditions. As the standard is normally applied to simulate industrial composting conditions at elevated temperatures (that are not occurring under home-composting conditions), the test temperature was reduced from 58 °C to 28 °C, which is in line with the suggested temperature of test programs on home compostability proposed by certification institutes TÜV Austria BELGIUM and DIN CERTCO. The test reactors were filled with 80 g of sample and 1200 g of inoculum and were incubated at 28 ± 2 °C. The inoculum used during the test was derived from organic municipal solid waste, which was composted under controlled aerated lab conditions for at least 20 weeks to a stable and mature compost. The used microorganism (bacteria and fungi)-rich inoculum itself was obtained after sieving (5 mm sieve) the mature compost (keeping the smallest fraction). All the used compost inocula had a total solid content of 50–55% and a volatile solid concentration higher than 30% in dry solids. The evolved CO_2_ was absorbed into 1 N KOH and determined by titration with hydrochloric acid (1 N) using a Metrohm 888 Titrando (Varese, Italy) and Tiamo™ 2.5 software (Varese, Italy).

#### 2.2.2. Soil Biodegradation Test (ISO 17556)

International standard ISO 17556 (Determination of the ultimate aerobic biodegradability of plastic materials in soil by measuring the oxygen demand in a respirometer or the amount of carbon dioxide evolved) (2019) was considered to test the biodegradation in soils. Standard soil (a mixture of 70% industrial quartz sand, 10% kaolinite clay, 16% natural soil and 4% mature compost) was used for the experiment. The used natural soil consisted of a mixture of 1/3 field soil (sandy field in Lokeren, Belgium) and 2/3 forest soil (2 types of forest in Moerbeke, Belgium). Before mixing, the collected natural soils were sieved to remove all the stones, roots, plant debris and other inert material. The mature compost was the same as described in ISO 14855 (2012). Additionally, a nutrient solution (per kg soil: KH_2_PO_4_ 0.2 g; MgSO_4_ 0.1 g; NaNO_3_ 0.4 g; urea 0.2 g; and NH_4_Cl 0.4 g/L) was added to the soil. At the beginning of the experiment, 2.0 g of the reference or test item was mixed with 500 g of prepared soil and incubated airtight in the dark at 25 ± 2 °C. The evolved CO_2_ was absorbed into 1 N KOH and determined by titration with hydrochloric acid (1 N) using a Metrohm 888 Titrando and Tiamo™ 2.5 software.

#### 2.2.3. Marine Biodegradation Test (ASTM D6691)

American standard ASTM D6691 (Standard Test Method for Determining Aerobic Biodegradation of Plastic Materials in the Marine Environment by a Defined Microbial Consortium or Natural Sea Water Inoculum) (2009) determines the biodegradation of a test item under controlled laboratory conditions by incubation in seawater. The reference and sample (approximately 60 mg) were added directly in a reactor containing 250 mL of enriched (0.05 g L^−1^ NH_4_Cl and 0.1 g L^−1^ KH_2_PO_4_) seawater. The natural seawater was collected from an open sea (Blankenberge, Belgium). The reactors were incubated at a constant temperature of 30 ± 2 °C in the dark. The CO_2_ produced was absorbed in a 3 N KOH solution and subsequently titrated with 0.05 N hydrochloric acid using a Metrohm 888 Titrando and Tiamo™ 2.5 software.

#### 2.2.4. Freshwater Biodegradation Test (ISO 14851)

International standard ISO 14851 (Determination of the ultimate aerobic biodegradability of plastic materials in an aqueous medium—Method by measuring the oxygen demand in a closed respirometer) (2005) was selected to simulate a freshwater environment. At the start, each reactor was filled 245 mL of mineral freshwater medium and 5 mL of inoculum. The inoculum was a mixture of activated sludge derived from various aerobic wastewater treatment plants in Belgium (Gent, Lokeren and Destelbergen). This activated sludge provided the microorganisms necessary for biodegradation. Each test reactor received 25 mg of the test or reference item. The reactors were incubated at a constant temperature of 21 ± 1 °C in the dark. After 310 days, the incubation temperature was increased to 30 ± 2 °C. Methods for CO_2_ measurement and biodegradation calculation followed ASTM D6691.

### 2.3. Field-Emission Scanning Electron Microscopy (FESEM)

The PLA/PCL and PLA/PBSA blends were cryofractured and sputtered with platinum before FESEM analysis. Hence, the fractured surfaces were analyzed to investigate the blend morphology using an FEI Quanta 450 FEG scanning electron microscope (SEM) (Thermo Fisher Scientific, Waltham, MA, USA). The sample surfaces were sputtered (on a LEICA EM ACE 600 High Vacuum Sputter Coater, Wetzlar, Germany) with platinum.

## 3. Results

In all tests, the biodegradation validity requirement for the reference item (cellulose) was met, achieving a biodegradation level of 70% under home-composting and marine conditions and 60% in a soil and freshwater environment, thereby confirming the validity of the experiments.

### 3.1. Results About Home-Composting Biodegradation

For a polymer to be considered home-compostable, it must reach a biodegradation threshold level of over 90% after 365 days of composting. Our results show that PLA alone is not home-compostable (Figure 1 and Figure 2, Table 3). The relative biodegradation percentages exceeding 100% indicate that the test items achieved higher biodegradation than the reference item, cellulose.

Our findings align with the literature and are not unexpected, as pure PLA biodegradation requires a thermal trigger above 50 °C to initiate [8,11,13]. Above 50 °C, which is PLA’s glass transition temperature, molecular motion within the polymer chain increases, causing PLA to become “rubbery”. This enhances water absorption, accelerating both hydrolysis and microbial attachment and thus making it more susceptible to biodegradation [11]. However, home-composting experiments were conducted at lower temperatures (in this case, 28 °C) and did not exhibit this effect. Our results indicate that low-crystalline PLA can become biodegradable under home-composting conditions when blended with specific ratios of PCL or PBSA. The PLA90_PCL10, PLA80_PCL20 and PLA50_PCL50 blends all achieved a relative biodegradation (the biodegradation level relative to the reference material) exceeding 90% within 365 days, the duration of a standard ISO 14,855 experiment. This meets the biodegradation requirements for home composting as outlined in Australian standard AS 5810, French standard NF T 51, and the home composability certification program of TÜV Austria Belgium and DIN CERTCO [33,34] (Figure 1, Table 3). For example, PLA80_PCL20 exceeded the relative biodegradation threshold of 90% after just 165 days of composting, closely matching observations made by Narancic et al. (2018) [8]. Although PLA95_PCL5 does not qualify as home-compostable within 365 days, it is noteworthy that this blend reached a relative biodegradation level of over 90% after 407 days. The biodegradation curve (Figure 1, red trend) shows a large value for the standard deviation due to a higher observed dispersion of values around the average, indicating that the degradation mechanism, with a very low amount of PCL, is less reliable and more affected by the local characteristics of the sample.

Among the PLA_PBSA blends tested, only the blend containing 60% PLA achieved the 90% biodegradation requirement within 365 days (Figure 2 and Table 3). Additionally, PLA80_PBSA20 reached the relative biodegradation threshold level only after 710 days of composting. These findings indicate that a higher PBSA content is needed to ensure a sufficiently rapid biodegradation rate for home compostability, especially when compared with PLA/PCL blends (Figure 3). Interestingly, the observed standard deviation for PLA_PBSA blends was lower than that for PLA/PCL blends (Figure 2), suggesting greater consistency in the biodegradation performance of PLA_PBSA blends.

The results also demonstrate that the biodegradation can be adjusted by varying the amount of the home-compostable biopolyester (BP) added, such as PCL or PBSA. Higher BP content led to a faster onset of biodegradation. For example, while biodegradation began after 30 days for PLA50_PCL50, it started about 10 days later for PLA80_PCL20, and another 10 days later for PLA90_PCL10. The blend with only 5% PCL began to degrade after about 150 days. A similar trend was observed for the PLA/PBSA blends. Since the lag phase was consistent across replicates, these findings suggest potential for the development of compounds with tailored biodegradation rates. Given that biodegradation rates also depend on environmental conditions, the biodegradation in soil and aquatic conditions was investigated.

### 3.2. Results of Soil Biodegradation Test

The degradation of plastics in soils is generally a complex and slower process compared to composting. This is mainly due to lower moisture content, a narrower temperature range and consequently reduced hydrolytic potential, as well as a lower abundance of microorganisms [35]. This phenomenon was also observed in the soil biodegradation tests for PLA90_PCL10 and PLA80_PCL20 (Figure 4), as well as for PLA60_PBSA40 (Figure 5).

While less than 10% biodegradation was observed for PLA80_PCL20 after 1 year, the biodegradation level gradually increased, resulting in a biodegradation of 43% after 3 years. This trend was mainly attributed to significant degradation in one of both replicates, which also explains the standard deviation observed (Figure 4). Since biodegradation did not progress in the other replicates, a cross-inoculation was performed between these replicates after 930 days, leading to the initiation of biodegradation in the previously inactive replicate (Figure 6). This indicates that the microbial population plays an important role in the degradation process. One replicate of PLA80_PCL20 reached 90% biodegradation after 1172 days. After three years, biodegradation for PLA90_PCL10 began in both replicates, while PLA60_PBSA40 exhibited biodegradation starting after one year, with an acceleration noted after 540 days.

### 3.3. Results of Marine and Freshwater Biodegradation Tests

The biodegradation of PLA LX175 and PLA60_PBSA40 was also investigated under marine pelagic conditions according to ASTM D6691 at a temperature of 30 °C (Figure 7). In contrast to testing under home-composting conditions and in soil, biodegradation was observed for PLA LX175 as well. Both materials began to show signs of biodegradation after 2 months. After 182 days of incubation under marine pelagic conditions at 30 °C, the absolute biodegradation measured was 11.7% for PLA LX175 and 38.2% for PLA60_PBSA40. Over time, the biodegradation of PLA accelerated compared to PLA60_PBSA40, reaching similar levels after 379 days (74% biodegradation). Both materials achieved a relative biodegradation percentage exceeding 90% after 448 for PLA LX175 and 406 days for PLA60_PBSA40.

The marine biodegradation test was conducted at 30 ± 2 °C, which corresponds to the maximum sea surface water temperature [36]. However, this temperature is relatively high, and under natural conditions, the biodegradation of plastics typically occurs at much lower temperatures, resulting in a slower degradation rate [11]. Using higher temperatures in laboratory experiments is a well-considered compromise, as testing under natural/colder conditions would require several years to yield results [37]. Nevertheless, this temperature difference should be considered when formulating plastic waste policies, as significantly lower biodegradation rates are expected at lower temperatures.

**Figure 8:**
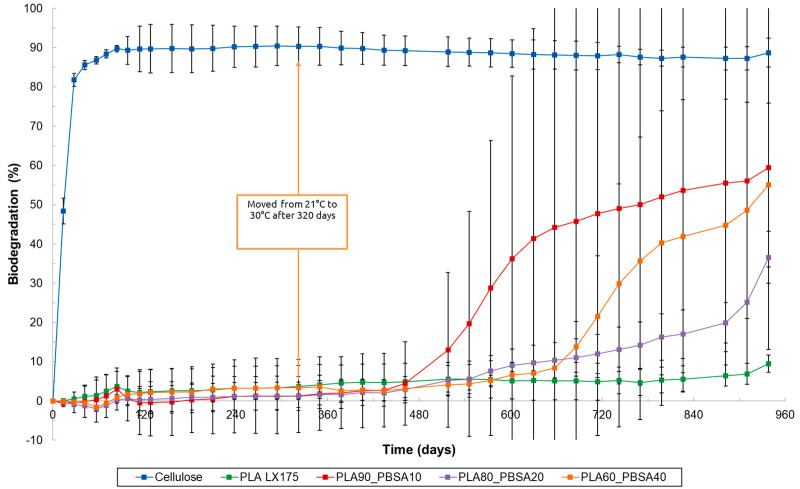
Biodegradation of cellulose (=reference material), PLA LX175 and PLA and PBSA plastic blends under aquatic freshwater conditions (ISO 14851, 21 °C).

Under aquatic freshwater conditions according to ISO 14851, at 21 °C a much slower biodegradation was observed (Figure 8). In fact, after 320 days, no biodegradation was detected for PLA or the tested PLA_PBSA blends. Subsequently, the temperature was increased from 21 °C to 30 °C, matching the temperature used in the marine biodegradation test. This change resulted in a slight increase in biodegradation, except for one replicate of PLA90_PBSA10, which experienced a significant increase in biodegradation after 462 days, reaching 89.6% after 798 days. It is likely that the microorganisms in this replicate adapted, leading to enhanced growth and consequently increased the degradation of PLA. A similar phenomenon was observed for PLA60_PBSA40 after 658 days, when one replicate exhibited a sharp increase in biodegradation, achieving 66.5% after 826 days, while the other replicate showed a more gradual increase, reaching only 17.4% at that time. The standard deviation for these freshwater experiments was notably large, suggesting reduced reliability in the behavior of all tested blends (Figure 8). In addition to differences in microbial population, the temperature difference between the marine (30 ± 2 °C) and freshwater (21 ± 1 °C) biodegradation tests likely contributed to the faster biodegradation of PLA and its blends under marine conditions. However, since the pure PLA also degrades relatively quickly under marine conditions—a trend not observed in home-composting, soil and freshwater (even not at 30 °C for 560 days) conditions—it can be inferred that the salts present in marine water may also influence the rate of hydrolysis and consequent degradation of PLA.

### 3.4. Correlations Between Processing, Phase Morphology and Biodegradation of PLA Blends

**Figure 9:**
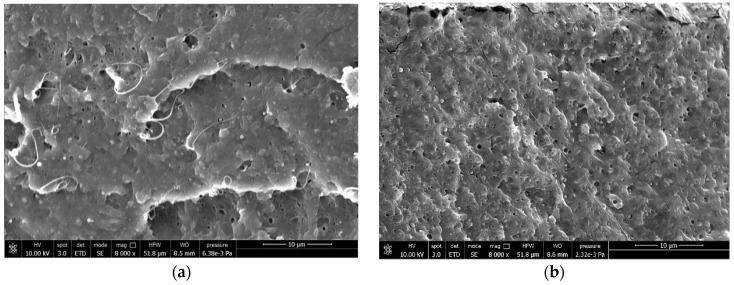

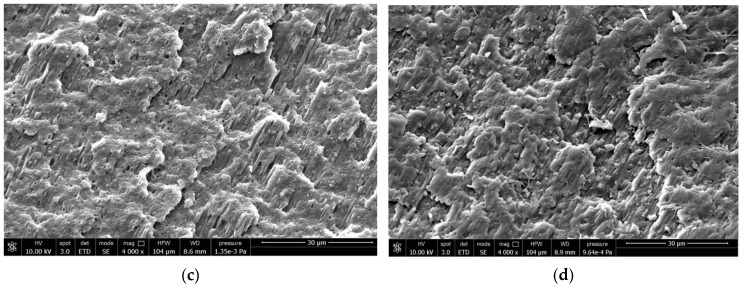
FESEM of (**a**) PLA95_PCL5, (**b**) PLA90_PCL10, (**c**) PLA80_PCL20 and (**d**) PLA50_PCL50 blends.

These biodegradation trials in different environments demonstrate that PLA with low crystallinity can become biodegradable at mild-temperature conditions by blending with PCL or PBSA. In order to better understand this behavior, the morphology of the PLA/PCL and PLA/PBSA blends was investigated by FESEM. In Figure 9, it is possible to observe the morphology of the PLA/PCL blends.

**Figure 10:**
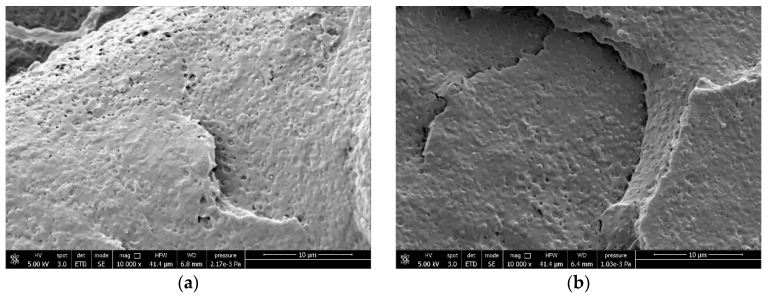

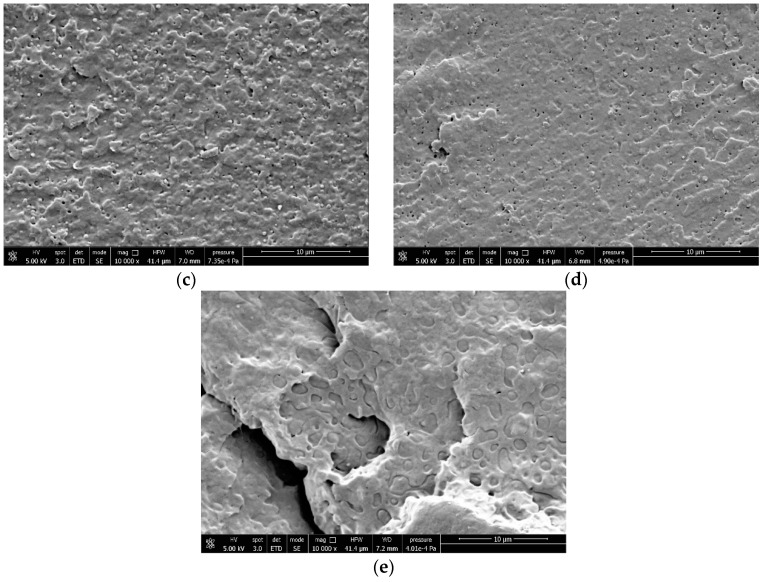
FESEM of (**a**) PLA95_PBSA5, (**b**) PLA90_PBSA10, (**c**) PLA85_PBSA15, (**d**) PLA80_PBSA20 and (**e**) PLA60_PBSA40 blends.

The granules of PLA containing PCL showed a dispersed morphology of up to 10% by PCL weight. Then, at 20% and up to 50%, they turned out co-continuous. Since at less than 20% of PCL, complete home-compostability could be predicted (Figure 3), the phase distribution is reasonably influencing the biodegradation rate. For the PLA/PBSA blends, a co-continuous phase distribution was attained only for the blend containing 40% of PBSA (Figure 10).

**Figure 11:**
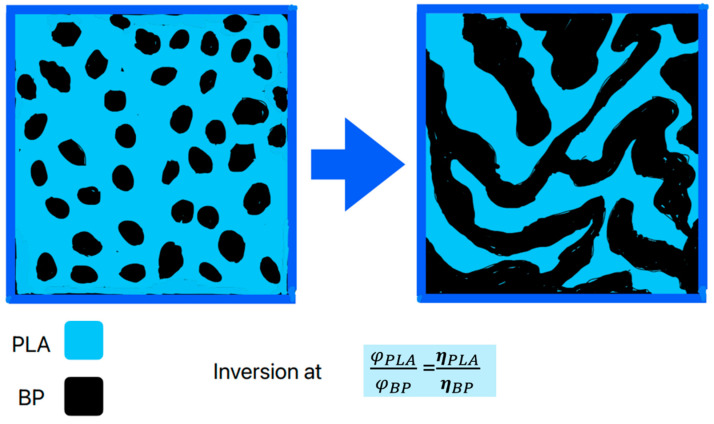
Representation of phase inversion composition in immiscible PLA blends, depending on viscosity ratio.

Both the polymer couples are immiscible, but they are characterized by a high compatibility, due to the fact that PLA, PCL and PBSA are all biopolyesters, so their chemical affinity is very high. Nevertheless, the phase inversion point depends on the ratio with viscosity (Figure 11), following the equation proposed by Jordhamo et al. (1986) [38]. In comparing the MFR values of pure polymers, as reported in Table 2, and considering that fluidity is inversely proportional to melt viscosity η, the polymers can be classified in terms of viscosity (deducted 2):

(1) $$\eta_{\mathrm{PLA}\;4043D}∼\eta_{\mathrm{PLA}\;\mathrm{LX}175}\;>\;\eta_{\mathrm{PBSA}}\;>\;\eta_{\mathrm{PCL}}$$

Hence, the ratio between viscosity is necessarily

(2) $$\frac{\eta_{PLA\;4043D}}{\eta_{PCL}}>\frac{\eta_{PLA\;LX175}}{\eta_{PBSA}}$$

and thus, considering Jordhamo et al.’s (1986) equation, the composition for phase inversion is higher in terms of PLA content for the PLA/PCL blend than for the PLA/PBSA blend:

(3) $$\frac{\varphi_{PLA\;4043D}}{\varphi_{PCL}}>\frac{\varphi_{PLA\;LX175}}{\varphi_{PBSA}}$$

Thus, the inversion point of PLA/PCL blends is at very low PCL contents because of the low viscosity of PCL. PCL and PBSA are highly degradable in home-composting conditions.

The PCL and PBSA phases are likely available for microbial attack and are degraded in the first step, by which acids and protons H+ are formed and can diffuse in the amorphous PLA structure. This initial breakdown allows water to enter the PLA, especially when the PCL or PBSA forms a phase co-continuous distribution. In fact, in co-continuous blends, the interpenetration of phases facilitates the diffusion of moisture in the PLA phase through the more degradable PCL and PBSA phases to start PLA hydrolysis. The acids can start the hydrolysis of the PLA with the formation of carboxyl groups that can then self-catalyze the degradation of PLA [11]. Consequently, relatively fast degradation can be established without increased temperature conditions (>50 °C). The first step, involving the degradation of PCL and PBSA and subsequent PLA hydrolysis, appears to be the most time consuming. In the tested environments, PCL and PBSA are degraded the fastest under home-composting conditions, explaining the faster degradation compared to soil and freshwater. Under marine conditions, PLA LX175 was degradable by itself and, as such, was less influenced by the biodegradation of PBSA, which only proceeds at a slow rate under marine conditions.

## 4. Conclusions

Over the past decade, public awareness of micro- and macro-plastics in the environment has increased significantly, underscoring the need for biodegradable plastics to replace persistent, non-degradable ones.

The present study clearly evidences that PLA could become more biodegradable when blended with other biodegradable polymers such as PCL and PBSA. Under home-composting conditions, all tested PLA/PCL blends (ranging from 10 to 50 wt.% PCL) and PLA60_PBSA40 were biodegraded within 365 days. Even blending PLA with as little as 5 wt.% PCL significantly improved its biodegradability, making it home-compostable after 407 days. However, biodegradation was considerably slower in other environmental conditions. For instance, although PLA60_PBSA40 is biodegradable under home-composting conditions, only slow biodegradation was observed in soil (25 °C) and freshwater (21 °C). In marine conditions at 30 °C, both PLA60_PBSA40 and pure PLA exhibited significant biodegradation rates, while freshwater proved to be the most challenging environment for PLA degradation. Hence, the present work evidences, in a comparative way, the different degradation behaviors of PLA and its blends in different contexts, suggesting a possible interpretation of the obtained results based on the phase morphology’s dependence on composition.

While it is fundamental to engage citizens in the responsible management of end-of-life products, it is evident that our findings highlight opportunities for developing applications in open environments, where the risk of the accidental dispersion of plastic is higher, such as tree trunk protectors, agricultural growth aids, fishing nets, geotextiles, etc. These applications should resist biodegradation during their functional life but ideally become biodegradable within a reasonable time frame afterward, addressing current environmental contamination from plastic products. In considering a long-term strategy for material design, increasing the biodegradability of biobased plastics across various environments is a crucial step toward enhancing the global sustainability of our planet and certainly warrants further research.

## Figures and Tables

**Table 1:** Characteristics of polymers used in this study.

Polymer	Origin	Trade Name	Supplier	Molecular Weight Mn (Da)	D Content (wt.%)	MFR ^a^ (2.16 kg)	MFR (190°, 2.16 kg)	MVR (190°, 2.16 kg)
**PLA**	Biobased	Luminy LX175	Total Corbion, Gorinchem, The Netherlands	84,410 [29]	4	6 at 210 °C 3 at 190 °C	2.7 ± 0.2	2.2 ± 0.2
**PLA**	Biobased	Ingeo 4043D	NatureWorks, Plymouth, MN, USA	106,000 [30]	4, 5-5	6 at 210 °C	1.9 ± 0.9	1.6 ± 0.9
**PCL**	Petro-chemical	Capa 6500	Ingevity, North Charleston, SC, USA	49,000 [31]	-	7 at 160 °C,	28.0 ± 5.0	26.0 ± 4.0
**PBSA**	Biobased	FD92 PM	PTT MCC Biochem Company, Bangkok, Thailand	48,000 [32]	-	4 at 190 °C	7.1 ± 0.7	7.0 ± 0.7

^a^ data from technical sheets of commercial products.

**Table 2:** Blend name, composition and biodegradation test standard performed.

Blend Name	PLA (wt.%)	PCL (wt.%)	PBSA (wt.%)	Biodegradation Standard
PLA	100	0	0	ISO 14855, ISO 17556, ASTM D6691, ISO 14851
PCL	0	100	0	NA
PBSA	0	0	100	NA
PLA95_PCL5	95	5	0	ISO 14855
PLA90_PCL10	90	10	0	ISO 14855, ISO 17556
PLA80_PCL20	80	20	0	ISO 14855, ISO 17556
PLA50_PCL50	50	50	0	ISO 14855
PLA90_PBSA10	90	0	10	ISO 14855, ISO 14851
PLA80_PBSA20	80	0	20	ISO 14855, ISO 14851
PLA60_PBSA40	60	0	40	ISO 14855, ISO 17556, ASTM D6691, ISO 14851

**Table 3:** Average absolute and relative biodegradation of PLA LX175, PBSA and several PLA/PBSA plastic blends under home-composting conditions (ISO 14855, 28 °C) after 364 days of incubation.

Blend Name	Absolute Biodegradation (%)	Relative Biodegradation (%)	Day > 90% Relative Biodegradation
PLA 4043D	1.2	1.3	NA
PCL *	90.1	104.3	117
PLA95_PCL5	78.8	80.7	407
PLA90_PCL10 **	97.7	100.7	180
PLA80_PCL20 **	101.5	104.5	165
PLA50_PCL50	90.2	92.3	300
PLA LX175	3.3	3.6	NA
PBSA ***	91.8	103.5	105
PLA90_PBSA10	2.9	3.3	NA
PLA80_PBSA20	46.0	51.4	720
PLA60_PBSA40	88.3	99.3	210

* test stopped after 195 days; ** test stopped after 310 days; *** test stopped after 210 days; NA: not applicable.

## Data Availability

The raw data supporting the conclusions of this article will be made available by the authors on request.
